# A case report and analysis of hypertrophic obstructive cardiomyopathy causing an illusion of aortic stenosis

**DOI:** 10.1097/MD.0000000000013711

**Published:** 2018-12-14

**Authors:** Ying Wang, Yue Ming Zhang, Jing Guang Dong, Li Jie Cheng, Guan Hua Jiang, Jian Wei Zheng, Wen Zhou Yu

**Affiliations:** a Department of Nursing, Weifang Medical College; b Heart Center, Sunshine Union Hospital, Weifang, Shandong procince, China.

**Keywords:** aortic stenosis, hypertrophic obstructive cardiomyopathy, misdiagnosis, ultrasound

## Abstract

**Rationale:**

This study aimed to report a case of hypertrophic obstructive cardiomyopathy causing an illusion of aortic stenosis on imaging.

**Patient concerns:**

A 71-year-old woman presented with chest tightness after activity for 1 year and coughing for 2 months. A systolic 3/6 grade murmur was found in the third intercostals of the left border of sternum. Transthoracic echocardiography, transesophageal echocardiography, and magnetic resonance imaging (MRI) were all suggestive of aortic stenosis and left ventricular outflow tract stenosis.

**Diagnosis:**

The patient was diagnosed with “severe aortic stenosis (bicuspid deformity), left ventricular outflow tract stenosis (moderate), and grade II cardiac function.“ She was advised aortic valve replacement and left ventricular outflow tract dredging. However, no aortic valve lesion was found during the operation, and the diagnosis was changed to “hypertrophic obstructive cardiomyopathy.”

**Interventions and outcomes:**

The morrow procedure was performed, and the patient recovered well after the operation. Hypertrophic obstructive cardiomyopathy was found to cause an illusion of aortic stenosis on imaging.

**Lessons:**

Special attention and rational treatment should be paid to such patients. In addition, further studies are needed to distinguish between the two diseases to reduce misdiagnosis.

## 1 Introduction

Aortic stenosis accompanied by secondary unsymmetrical septal hypertrophy had an incidence of about 10%,^[1,2]^ and aortic stenosis accompanied by primary hypertrophic obstructive cardiomyopathy was rare.^[3]^ Whether and how hypertrophic obstructive cardiomyopathy could affect the aortic valve movement was not reported. The applicability of cardiac magnetic resonance imaging (MRI) in assessing valvular heart disease is growing and being recognized in recent guidelines. Cardiac MRI has the ability to assess valve morphology and qualify and quantify valvular diseases.^[4–7]^ It is a useful technique in evaluating patients with hypertrophic cardiomyopathy and is preferred over other imaging modalities.^[8,9]^ The present study revealed that hypertrophic obstructive cardiomyopathy could cause an illusion of aortic stenosis on imaging (cardiac MRI and transesophageal echocardiography). Cardiac physicians should always be vigilant and respond rationally to such patients. In addition, further studies on imaging are needed to distinguish between the two diseases and reduce misdiagnosis.

## 2 Case report

**Figure 1:**
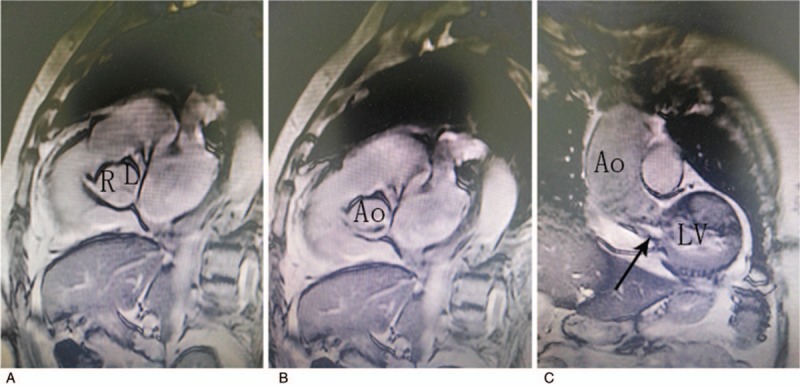
Magnetic resonance images. (A) Three leaflets were well closed when the aortic valve was closed. (B) The aortic valve was found with restricted opening, and the blood flow only pushed through the aortic noncoronary leaflet when the left ventricle was contracted. (C) The arrow shows that the white region was the high-velocity blood flow region when the left ventricle contracted, and only one leaflet (noncoronary leaflet) was pushed open.

**Figure 2:**
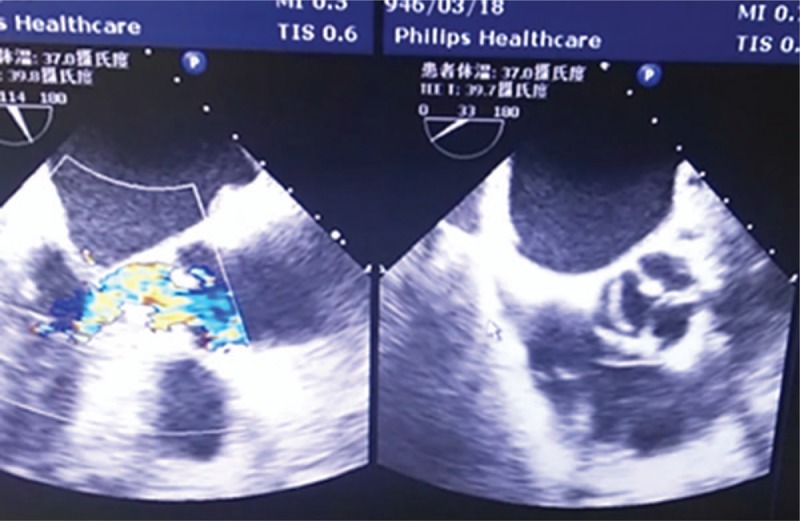
Long axis of transesophageal echocardiography shows hypertrophic obstructive cardiomyopathy with left ventricular outflow tract stenosis of a maximum thickness of 22 mm. The short axis shows that the noncoronary leaflet of aortic valve was well open in the systole, while the left and right coronary leaflets show restricted opening.

A 71-year-old elderly woman was admitted to Shandong Sunshine Union Hospital, Shandong, China, on November 10, 2017, due to chest tightness and breathing shortness after activity for 1 year and coughing for 2 months. The patient began to have chest tightness and breathing shortness after general physical activity 1 year ago and coughing (dry coughs mainly) 2 months ago. A small amount of white sputum was noted after a severe cough, which was heavy at night and light in the daytime. The patient was diagnosed with “severe aortic stenosis” using color Doppler echocardiography at several hospitals, and was admitted to the aforementioned hospital with “severe aortic stenosis (bicuspid deformity) and left ventricular outflow tract stenosis,” which was confirmed using transthoracic echocardiography. The patient had a history of hypertension for 10 years and usually took antihypertensive drugs to control blood pressure. The physical examination revealed a clear respiratory sound of bilateral lungs without rhonchus and moist rales and a systolic 3/6 grade murmur in the third intercostals of the left border of sternum. The liver was not enlarged and had no swelling in both lower extremities. The laboratory examination of brain natriuretic peptide was normal. Transthoracic echocardiography showed asymmetric hypertrophy of the left ventricle with a maximum thickness of 20 mm, the diameter of the left ventricular outflow tract of 13 mm, and the anterior flow velocity of the aortic valve of 4.3 m/s. Cardiac MRI revealed myocardial hypertrophy, left ventricular outflow tract stenosis, aortic stenosis, and bicuspid deformity (Fig. 1), indicating the requirement of aortic valve replacement and left ventricular outflow tract dredging.^[10]^ Intraoperative transesophageal echocardiography (Fig. 2) showed aortic stenosis and left ventricular outflow tract stenosis. Surprisingly, the aortic valve was normal without stenosis or insufficiency, and the asymmetrically hypertrophic myocardium under the aortic valve caused left ventricular outflow tract stenosis. Therefore, the diagnosis was changed to hypertrophic obstructive cardiomyopathy. The Morrow procedure was performed to remove the hypertrophic myocardium from the left side of the middle point of the right coronary leaflet to the left coronary leaflet. After the operation, the transesophageal echocardiography examination revealed that the velocity of left ventricular outflow tract decreased to 2.6 m/s and the opening restriction of aortic valve had been corrected (Figure unreserved). Hypertrophic cardiomyocytes were found in the pathology. The conditions of left ventricular outflow tract and aortic valve were observed using echocardiography in the follow-ups 6 months and 1 year after the operation.

## 3 Discussion

The patient was diagnosed with severe aortic stenosis at several hospitals; both cardiac MRI and transesophageal echocardiography at Shandong Sunshine union hospital also revealed aortic valve stenosis. Moreover, the patient was found to have left ventricular hypertrophy and outflow tract stenosis. Therefore, she was diagnosed with “severe aortic stenosis (bicuspid deformity) and left ventricular outflow tract stenosis.” Aortic stenosis was a primary lesion, and cardiac hypertrophy and left ventricular outflow tract stenosis were secondary changes. No abnormal aortic valve was found during the operation, and the diagnosis was changed to “hypertrophic obstructive cardiomyopathy. ” After a careful study of MRI scans of blood flow, dynamic images of transesophageal echocardiography, and intraoperative data, the aortic valve was found with restricted opening, although the aortic valve had no lesion. The reason for this stenosis was the asymmetrically hypertrophic myocardium under the aortic valve (the thickened myocardium was located mainly under the left and right coronary leaflets), causing the eccentric blood flow of the left ventricular outflow tract to shoot the noncoronary leaflet of aortic valve (Fig. 1). High-velocity eccentric blood flow was shot to the noncoronary leaflet, and was thus opened during the early stage of left ventricular systole. The right and left coronary leaflets were obstructed by hypertrophic myocardium, and thus not opened. The high-speed blood flow passed through the noncoronary leaflet into the aortic greater curvature, and then the flow was divided into two parts. One part continued to move forward, and the other part bent to the aortic lesser curvature and the left and right coronary leaflets of aortic valve. The right and left coronary leaflets were not opened because of the counterforce of the eddy current in the aorta caused by the initial high-velocity blood flow. Therefore, aortic stenosis could be found on the images of transthoracic echocardiography, transesophageal echocardiography, and cardiac MRI. Jeffrey et al thought that the difference in the degree of decreased pressure in the aorta between hypertrophic obstructive cardiomyopathy and aortic valve stenosis could be used to distinguish between the 2 diseases. However, this was unsuitable in the present case,^[11]^ which undoubtedly increased the difficulty in identifying this disease.

Thus, it is believed that hypertrophic obstructive cardiomyopathy could cause an illusion of aortic valve stenosis on imaging; therefore, both cardiologist and doctors from the ultrasonography department should consider this problem while making a diagnosis. The cardiac surgeon should be fully prepared before the operation, and doctors from the ultrasonography department should be careful to observe the movement of aortic valve from all angles and judge whether any lesion is present in the aortic valve so as to provide more information about patients with myocardial hypertrophy.

## Author contributions

**Conceptualization:** Wenzhou Yu.

**Formal analysis:** Yueming Zhang, Guanhua Jiang, Jianwei Zheng, Wenzhou Yu.

**Investigation:** Jingguang Dong, Lijie Cheng.

**Writing – original draft:** Ying Wang.

**Writing – review & editing:** Ying Wang.
